# Comparison between topping-off technology and posterior lumbar interbody fusion in the treatment of chronic low back pain: A meta-analysis

**DOI:** 10.1097/MD.0000000000018885

**Published:** 2020-01-31

**Authors:** Wei Wang, Xiangyao Sun, Tongtong Zhang, Siyuan Sun, Chao Kong, Junzhe Ding, Xiangyu Li, Shibao Lu

**Affiliations:** a Department of Orthopedics, Xuanwu Hospital Capital Medical University; b National Clinical Research Center for Geriatric Diseases; c Capital Medical University, China; d Department of Orthopaedics, ChuiYangLiu Hospital affiliated to Tsinghua University; e Department of Interdisciplinary, Life Science, Purdue University.

**Keywords:** interbody fusion, lumbar degenerative disease, meta-analysis, topping-off technique

## Abstract

**Background:**

The difference between topping-off technique and posterior lumbar interbody fusion (PLIF) in postoperative outcomes is still controversial. The aim of this study is to compare all available data on outcomes of topping-off technique and PLIF in the treatment of chronic low back pain.

**Methods:**

Articles in PubMed, EMBASE and Cochrane were reviewed. Parameters included radiographical adjacent segment disease (RASD), clinical adjacent segment disease, range of motion (ROM), global lumbar lordosis (GLL), visual analog scale (VAS), visual analog scale of back, (VAS-B) and visual analog scale leg (VAS-L), Oswestry disability index, Japanese Orthopaedic Association (JOA) score, duration of surgery, estimated blood loss (EBL), reoperation rates, complication rates.

**Results:**

Rates of proximal RASD (*P* = .001) and CASD (*P* = .03), postoperative VAS-B (*P* = .0001) were significantly lower in topping-off group than that in PLIF group. There was no significant difference in distal RASD (*P* = .07), postoperative GLL (*P* = .71), postoperative upper intervertebral ROM (*P* = .19), postoperative VAS-L (*P* = .08), DOI (*P* = .30), postoperative JOA (*P* = .18), EBL (*P* = .21) and duration of surgery (*P* = .49), reoperation rate (*P* = .16), complication rates (*P* = .31) between topping-off group and PLIF.

**Conclusions:**

Topping-off can effectively prevent the adjacent segment disease from progressing after lumbar internal fixation, which is be more effective in proximal segments. Topping-off technique was more effective in improving subjective feelings of patents rather than objective motor functions. However, no significant difference between topping-off technique and PLIF can be found in the rates of complications.

## Abstract 2

Supplemental Digital Content is available in the text

## 1 Introduction

Posterior lumbar interbody fusion (PLIF) has been considered to be the standard surgical treatment for patients suffering from chronic low back pain (CLBP) caused by lumbar degenerative disease.^[1]^ In addition to its satisfactory clinical outcomes, there are still a series of complications in PLIF.^[2]^ The increase in the range of motion (ROM) of adjacent segments will cause the acceleration of adjacent segment diseases (ASDs), which are the most commonly seen complications in the follow-up.^[3]^ Posterior dynamic stabilization systems, including interspinous process device (IPD); pedicle-based stabilization devices (PDS); total facet replacement system, have been used to decrease ASDs after PLIF since 1980 s.^[4]^ The most commonly used hybrid dynamic stabilization system is “topping-off ” technique. This technique combines the rigid fusion with dynamic nonfusion of adjacent segments, such as IPD or PDS, in order to reduce the hypermobility and overstress of the adjacent intervertebral disks.^[5–9]^ At the present study, there have been various flexible systems in spinal motion preservation technology, including PDS systems and IPS systems. The most commonly used PDS systems include Dynesys,^[10]^ NFlex,^[11]^ Isobar TTL,^[12]^ CD Horizon^[5]^ and DSS.^[13]^ Furthermore, the widely used IPS systems include Coflex,^[14]^ DIAM,^[15]^ Wallis.^[16]^

Because there is a lack of clear clinical evidences, the difference between topping-off technique and PLIF in postoperative outcomes is still controversial.^[9,17,18]^ Therefore, a meta-analysis was carried out in this study to compare all available data on postoperative clinical and radiographic outcomes of topping-off technique and PLIF in the treatment of CLBP.

## 2 Materials and methods

### 2.1 Search strategy

The present review was conducted according to preferred reporting items for systematic reviews and meta-analyses statement.^[19]^ An experienced librarian (XYL) carried out a comprehensive literature search. Relevant studies in PubMed, EMBASE, Cochrane databases from 1980 to October 2019 were identified. The medical subject headings and keywords included: ( [hybrid stabilization] or [topping off] or [hybrid stabilization device] or [dynamic hybrid] or [hybrid fixation] ) and ( [lumbar] or [lumbar degenerative disease] or [adjacent segment degeneration] or [ASD] and [fusion] ). Manual searches of all retrieved research and review reference lists were used to supplement the computer searches.

### 2.2 Inclusion criteria and exclusion criteria

The inclusion criteria included: clinical evaluation was followed up for no less than 1 year; conservative treatment was frustrated on the treatment of CLBP; PLIF or topping-off surgery was used in the treatment; patients were in the same preoperative radiographic baseline. Exclusion criteria included: biomechanical studies and non - human or in vitro studies; abstracts, case reports, expert opinions, and non-comparative studies; therapies for tumors, infections, revision surgeries or congenital malformations.

### 2.3 Data extraction and quality assessment

All data was extracted from the text, pictures, and tables of the articles by 2 authors (SYS, JZD). The data included: age, gender, duration, study design, enrolled number, radiographical adjacent segment disease (RASD) and clinical adjacent segment disease (CASD), ROM, global lumbar lordosis (GLL), visual analog scale (VAS) of back and leg (VAS-B, VAS-L), Oswestry disability index (ODI), Japanese Orthopaedic Association (JOA) score, duration of surgery, estimated blood loss (EBL), reoperation rates, complication rates. If there is a disagreement about the outcomes, other authors (WW, SBL) would participate in the discussion to reach a consensus. Newcastle-Ottawa scale (NOS)^[20]^ was used to assess the quality of the included studies by 2 authors (XYS, CK). A full score of 9 stars and a score of 7 or more is an excellent quality study.

### 2.4 Data analysis

The statistical analysis of the studies was performed in RevMan5.3 software. Odds ratios (OR) and standardized mean difference (SMD) with 95% confidence intervals (CI) were used for dichotomous data and continuous data. Heterogeneity across trials were explored according to the results of Chi-squared test and *I*^2^ statistic. Random-effects model was considered if there was a significant heterogeneity assumed as *P* value less than .05 and *I*^2^ > 50%. Otherwise, data were pooled by using the fixed-effects model. If there was a potential heterogeneity, subgroup analysis and sensitivity tests would be performed in conjunction with possible clinical realities. Publication bias was analyzed by the funnel plot.

## 3 Results

### 3.1 Study characteristics

**Figure 1:**
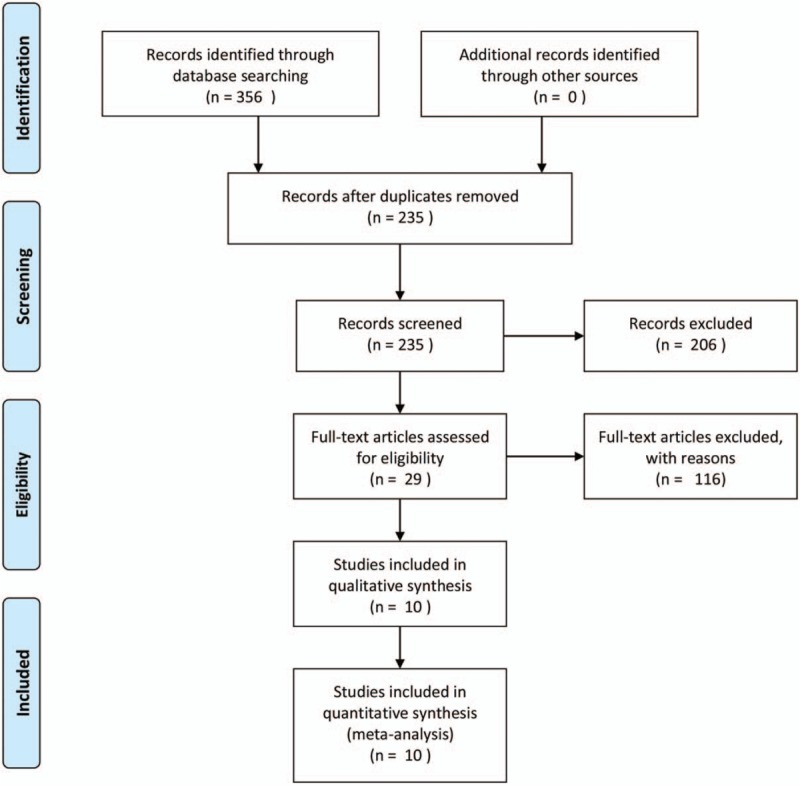
Flow chart showing identification and selection of cases.

A total of 356 articles were identified in the initial examination. After exclusion of duplicate or irrelevant articles, 256 articles were retrieved. Ultimately, 10 studies^[7,14,15,21–27]^ were finally included in the meta-analysis (Fig. 1). Table 1 showed the characteristics of the included studies. Table 2 showed the results of NOS.

**Table 1:**
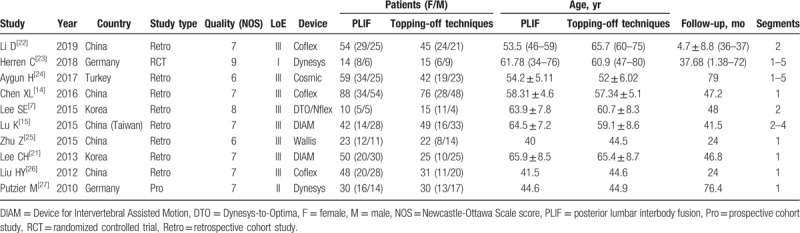
Characteristics of included studies.

**Table 2:**
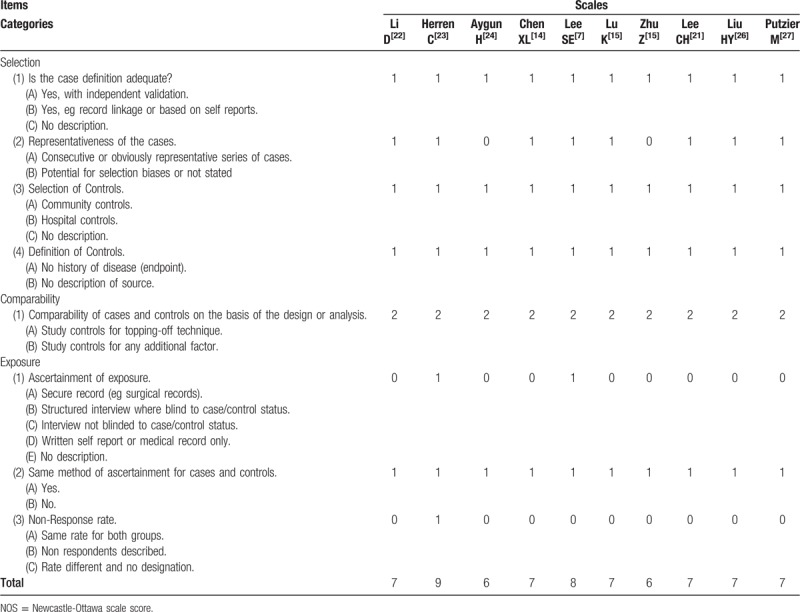
Newcastle-Ottawa scale (NOS).

### 3.2 ASD

**Figure 2:**
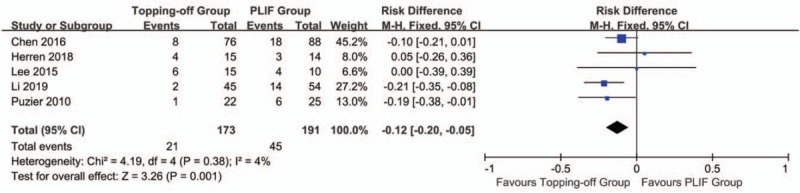
Forest plot of proximal RASD.

**Figure 3:**
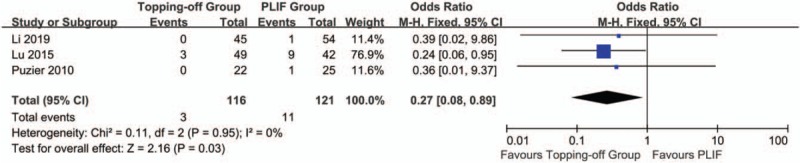
Forest plot of CASD.

Five studies^[7,14,22,23,27]^ reported the incidence of proximal RASP (Fig. 2). Considering there is no significant heterogeneity between 2 groups, the fixed effect model was applied (*I*^2^ = 4%). The incidence of proximal RASD in topping-off group was significantly less than that in PLIF group (OR -0.12; 95% CI -0.20, -0.05; *I*^2^ = 4%; *P* = .001). The funnel plot showed no significant publication bias (Supplementary File 1 The incidence of distal RASD was discussed in 2 studies.^[7,14]^ Because there is no significant heterogeneity between 2 groups (*I*^2^ = 5%), fixed effect model was applied in this analysis. No significant between-group difference was found in this analysis (OR 0.27; 95% CI 0.07, 1.11; *I*^2^ = 9%; *P* = .07). Three articles^[15,22,27]^ reported the incidence of CASD. The fixed effect model was applied considering there is no significant heterogeneity between 2 groups (*I*^2^ = 0%). The incidence of CASD in topping-off group was significantly less than that in PLIF group (OR 0.27; 95% CI 0.08, 0.89; *I*^2^ = 0%; *P* = .03) (Fig. 3). The funnel plot showed no significant publication bias (Supplementary File 2).

### 3.3 Radiographic parameters

**Figure 4:**
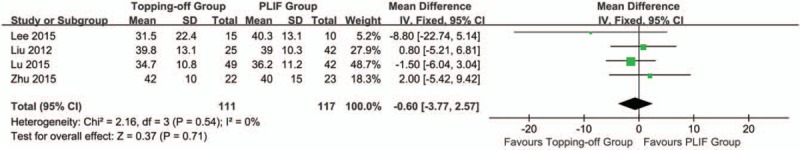
Forest plot of postoperative GLL. GLL = global lumbar lordosis.

Postoperative GLL was discussed in 4 studies.^[7,15,25,26]^ Considering there is no significant heterogeneity between 2 groups, the fixed effect model was applied (*I*^2^ = 0%). No significant between-group difference was found in fixed-effects model (SMD -0.60; 95% CI -3.77, 2.57; *I*^2^ = 0%; *P* = .71, Fig. 4). The funnel plot showed no significant publication bias (Supplementary File 3).

**Figure 5:**
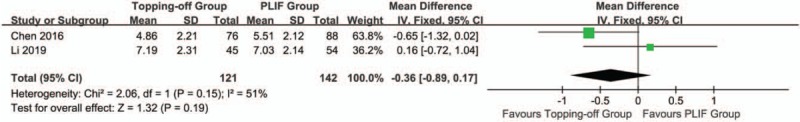
Forest plot of ROMs of intervertebral levels. ROMs = range of motions.

ROMs of upper intervertebral levels were discussed in two articles.^[14,22]^ The fixed effect model was applied considering there is no significant heterogeneity between two groups (I^2^ = 51%). No significant between-group difference was found in this analysis (SMD -0.36; 95% CI -0.89, 0.17; *I*^2^ = 0%; *P* = .71, Fig. 5)

### 3.4 Clinical scoring system

**Figure 6:**
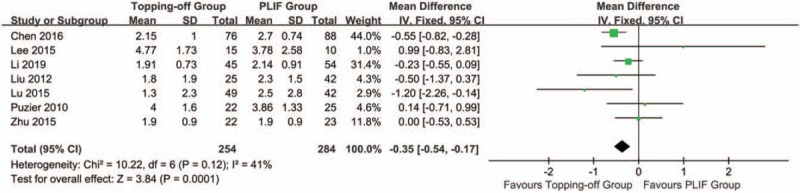
Forest plot of postoperative VAS-B. VAS-B = VAS of back.

VAS-B was documented in 7 articles^[7,14,15,25–27]^ (Fig. 6). Because there is no significant heterogeneity between 2 groups, the fixed effect model was applied (*I*^2^ = 41%). VAS-B in the Topping-off group was significantly less than in the PLIF group (SMD -0.35; 95% CI -0.54, -0.17; *I*^2^ = 41%; *P* = .0001). Funnel plot showed no significant publication bias in these studies (Supplementary File 4). Three studies^[7,15,22]^ evaluated VAS-L. Fixed effect model was used in this analysis (*I*^2^ = 65%). No significant between-group difference was found in this analysis (SMD -0.21; 95% CI -0.45, 0.02; *I*^2^ = 65%; *P* = .08). The funnel plot showed no significant publication bias (Supplementary File 5).

**Figure 7:**
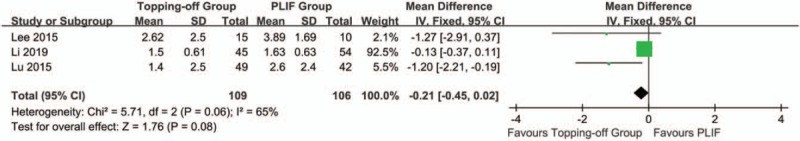
Forest plot of postoperative VAS-L. VAS-L = VAS of leg

**Figure 8:**
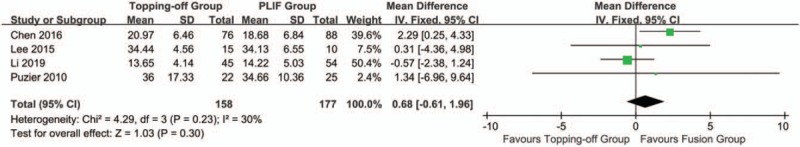
Forest plot of postoperative ODI. ODI = Oswestry disability index.

Four studies^[7,14,22,27]^ reported postoperative ODI. No significant heterogeneity (*I*^2^ = 30%) was found. The fixed-effect model was used in this analysis. No significant between-group difference was found in this analysis (SMD 0.68; 95% CI -0.61, 1.96; *I*^2^ = 30%; *P* = .30) (Fig. 8). The funnel plot showed no significant publication bias (Supplementary File 6). 2 studies^[25,26]^ discussed postoperative JOA. No between-group difference could be found in this analysis (SMD -0.90; 95% CI -2.22, 0.42; *I*^2^ = 0%; *P* = .18).

### 3.5 Intraoperative parameters

**Figure 9:**
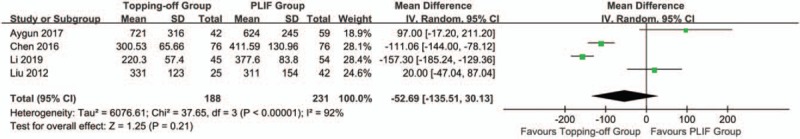
Forest plot of EBL. EBL = estimated blood loss.

**Figure 10:**
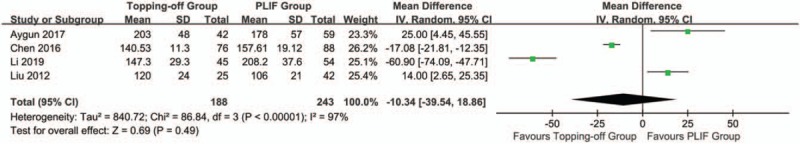
Forest plot of duration of surgery.

EBL was reported in 4 studies.^[14,22,24,26]^ Random effect model was used in this analysis, because a significant heterogeneity could be found (*I*^2^ = 92%). No between-group significance could be found in EBL (SMD -52.69; 95% CI -135.51, 30.13; *I*^2^ = 92%; *P* = .21) (Fig. 9). Duration of surgery was documented in 4 studies.^[14,22,24,26]^ Random effect model was used in this analysis, because there was a significant heterogeneity between these studies (*I*^2^ = 97%). There was no significant difference between these studies (SMD -10.34; 95% CI -39.54, 18.86; *I*^2^ = 97%; *P* = .49) (Fig. 10).

### 3.6 Complications

**Figure 11:**
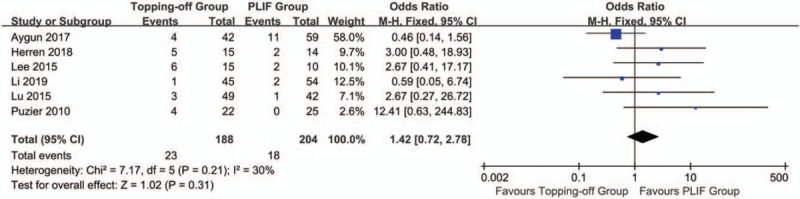
Forest plot of complication rates.

Six studies^[7,15,22–24,27]^ reported incidences of complications. Fixed effect model was used in this analysis without a significant heterogeneity (*I*^2^ = 30%). No publication bias could be found in this evaluation (Supplementary File 7). No significant between-group difference could be found in the results (OR 1.54; 95% CI 0.76, 3.11; *I*^2^ = 40%; *P* = .23) (Fig. 11).

Dural tear rates was reported in 2 studies.^[15,24]^ Fixed effect model was used in this analysis (*I*^2^ = 0%). No significant difference was found between topping-off group and PLIF group (OR 1.89; 95% CI 0.24, 15.10; *I*^2^ = 0%; *P* = .55). Two studies discussed the infection rates.^[15,24]^ No significant between-group difference could be found in the results (OR 0.64; 95% CI 0.08, 5.11; *I*^2^ = 0%; *P* = .67). Three articles^[7,24,27]^ discussed the pseudoarthrosis rates. Fixed effect model was used in this analysis (*I*^2^ = 0%). No between-group significance could be found in the results (OR 1.09; 95% CI 0.36, 3.27; *I*^2^ = 0%; *P* = .88) (Fig. 13).

**Figure 12:**
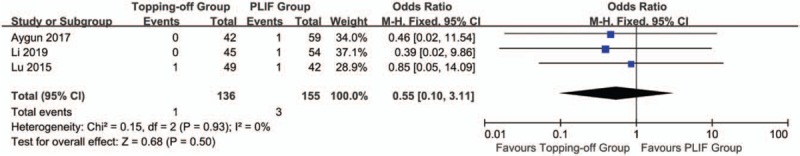
Forest plot of infection rates.

**Figure 13:**
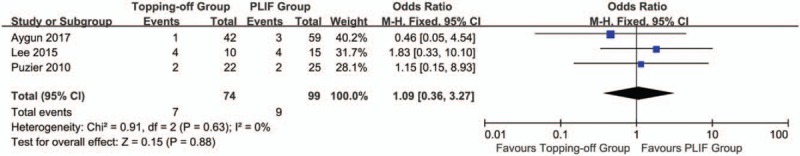
Forest plot of pseudoarthrosis rates.

**Figure 14:**
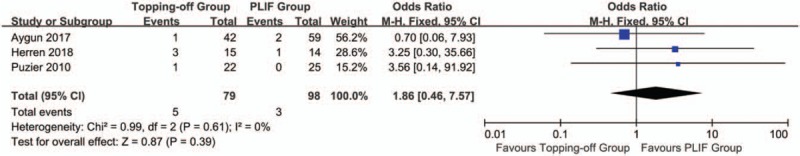
Forest plot of screw loosening rates.

Incidences of screw loosening were discussed in 3 articles.^[23,24,27]^ Fixed effect model was used in this evaluation (*I*^2^ = 0%) and no significant difference was found between topping-off group and PLIF group (OR 1.86; 95% CI 0.46, 7.57; *I*^2^ = 0%; *P* = .39) (Fig. 14). The funnel plot showed no significant publication bias (Supplementary File 8). Two articles^[23,27]^ discussed implant breakage rates. There was no significant difference between topping-off group and PLIF group (OR 2.15; 95% CI 0.39, 11.81; *I*^2^ = 57%; *P* = 0.38).

**Figure 15:**
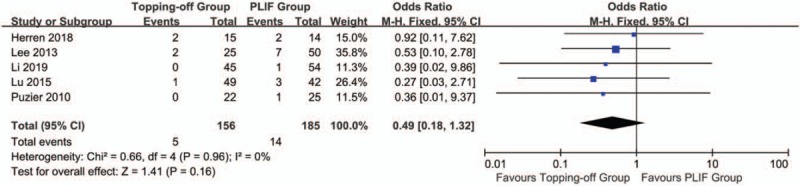
Forest plot of reoperation rates.

Five studies discussed reoperation rates.^[15,21–23,27]^ There was no significant heterogeneity in this analysis (*I*^2^ = 0%). Fixed effect model was used in this evaluation. No significant between-group difference was found in the results (OR 0.49; 95% CI 0.18, 1.32; *I*^2^ = 0%; *P* = .16) (Fig. 15). Funnel plot showed there was no significant publication bias in these studies (Supplementary File 9).

### 3.7 Age

**Figure 16:**
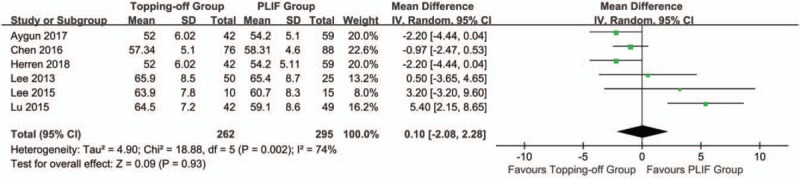
Forest plot of age.

Six studies discussed age.^[7,14,15,21,23,24]^ Random-effects model was used in this evaluation, because there was a significant heterogeneity in this analysis (*I*^2^ = 74%). No significant between-group difference was found in the results (SMD 0.10; 95% CI -2.08, 2.28; *I*^2^ = 74%; *P* = .93) (Fig. 16).

### 3.8 Gender

**Figure 17:**
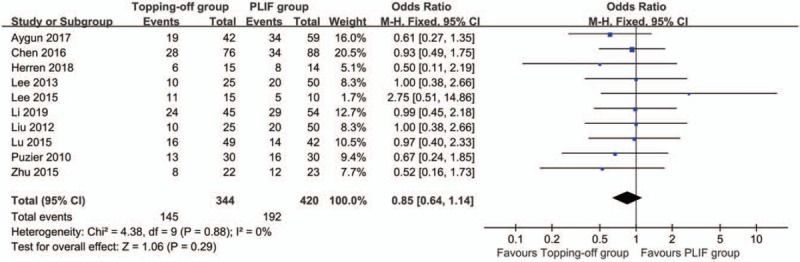
Forest plot of gender.

The influences of gender on the clinical outcomes of topping-off group and PLIF group were reported in 10 studies.^[7,14,15,21–27]^ There was no significant heterogeneity in this analysis (*I*^2^ = 0%). Fixed effect model was used in this evaluation. No significant between-group difference was found in the results (OR 0.85; 95% CI 0.64, 1.14; *I*^2^ = 0%; *P* = .29) (Fig. 17). Funnel plot showed there was no significant publication bias in these studies (Supplementary File 10).

## 4 Discussion

It is still controversial that whether ASD is a natural evolution of an aging spine or a consequence of spinal fusion.^[28,29]^ Previous studies stated that the risk factors for ASD included age over 50 years,^[30,31]^ sagittal imbalance,^[32]^ increased length of fusion and surgical approaches.^[33]^ The elastic fixation in topping-off technique can act as a partially active buffer between fused segments and proximal mobile segments. Therefore, the ASD could be reduced by topping-off technique via the application of dynamic fixation, which could prevent the proximal adjacent segments from degenerating.^[34]^

Because there is still a lack of uniformity in the criteria of RASD, its definition in the previous studies were summarized: spondylolisthesis increase more than 3 mm, loss of intervertebral disc height, dynamic angulation of the interspinous space less than 10°.^[7,14,15,21,24,25,35]^ Considering that there is no significant heterogeneity in the analysis of RASD and CASD in our results, our research has good reference value. Our study showed that the incidences of proximal RASD and CASD in the PLIF group were significantly higher than those in topping-off group. The results of this study are consistent with previous studies.^[9,36]^ However, the result of distal RASD rate showed no significant between-group difference. This implied that topping-off technique was more effective in preventing proximal ASD. The possible explanation is that the compensatory mechanism of ROM in the proximal segments in the topping-off group can be improved.^[34,37]^ Our results showed that No significant between-group difference was found in ROMs of upper intervertebral levels. Therefore, the decrease of compensatory mechanism in topping-off technique may work through multiple segments. The postoperative GLL was similar between topping-off group and PLIF group in our results. This indicates that the expansion effect in topping-off technology can be negligible in global spine compared with PLIF. However, the correction of GLL may not be an advantage of topping-off technology.^[23,34]^

The recovery of lumbar functions and the curative effects of operation were evaluated by JOA score, ODI and VAS. Our results showed that VAS-B in the topping-off group was significantly less than that in the PLIF group; however, no significant between-group difference was found in VAS-L. This showed that topping-off technique was more effective in relieving CLBP. However, JOA score and ODI were similar between both groups. The possible explanation is that scoring systems are associated with the increasing age and the complications of dynamic equipment.^[38]^ VAS gives more weight to subjective feelings of patents, while JOA score and ODI are more focused on the objective motor functions of patients.^[25,26]^ All of these indicate that topping-off technique, compared with PLIF, is more effective in improving subjective feelings of patents rather than objective motor functions. This conclusion is in consistent with the founding of previous studies, which showed that topping-off technique could achieve a good clinical improvement even in the long-term follow-up.^[4,39,40]^

Our study showed that no significant difference was found in EBL and duration of surgery between topping-off group and PLIF. The possible explanation is that additional exposure of anatomical structures is not needed in the insertion of dynamic implants; this will save the operation time.^[4]^ Considering there are many confounding factors in the included studies, this result needs to be interpreted carefully.

Whether topping-off technique can decrease the incidence of complications after fusion surgery or not has been inconclusive. The most commonly seen postoperative complications in topping-off technique are screw loosening, screw fracture and spinous process avulsion fracture.^[15,21,23,27]^ Screw loosening is most likely to be found in Hybrid Stabilization Devices; in addition, spinous process fracture is the most commonly seen complication in Interspinous Process Devices.^[41,42]^ The rates of complications, such as dural tear, infection, implant loosening, pseudoarthrosis, and implant breakage, were discussed in our study. However, no significant difference between topping-off group and PLIF group was found in our results. The “halo zone” in dynamic stabilization systems show that the forces conveyed from the dynamic implant can increase the stress on rigid fixation over time, and then implant-associated adverse events will occur.^[38]^ However, our study indicated that, compared with PLIF, the application of the topping-off technique would not be influenced by this effect. The application of hydroxyapatite coated pedicle screws might be an ideal method to prevent implant related complications.^[43]^ Previous studies showed that hydroxyapatite could promote bone deposition on the implant surface and promote the formation of direct chemical bonds between the implant and the bone interface, which might reduce the complication rates in patients.^[28,44]^

It has been reported that ASD after PLIF is not a complication caused by the fusion itself; ASD is more likely to be cause by normal aging process.^[45]^ However, Lu et al.^[15]^ reported that different methods of surgical treatments had greater influences on ASD than age and gender. Similarly, our results showed that no significant between-group difference was found in age and gender. This indicated that age and gender have a relatively smaller influence on the outcomes of the surgery.

There are several limitations in this meta-analysis. First, lumbar degeneration was a series of diseases in which the overall outcome could vary depending on specific diagnosis, such as intervertebral disc herniation, stenosis, and spondylolisthesis. Second, most of the included studies were retrospective studies. As a result, there would be an inherent limitation associated with the risks of reporting or selection bias. Third, different types of dynamic devices used in adjacent segments could have different structures, which might influence the outcomes. Therefore, more randomized controlled trials were needed to verify the results of this study.

## 5 Conclusions

Topping-off can effectively prevent the ASD from progressing after lumbar internal fixation, which is be more effective in proximal segments. Compared with PLIF, topping-off technique was more effective in improving subjective feelings of patents rather than objective motor functions. However, no significant difference between topping-off technique and PLIF can be found in the rates of complications.

## Acknowledgments

This research was performed mainly at the Department of Orthopaedics of Xuanwu Hospital Capital Medical University and in the National Clinical Research Center for Geriatric Diseases.

## Author contributions

**Conceptualization:** Wei Wang, Xiangyao Sun, Tongtong Zhang, Xingyu Li.

**Data curation:** Wei Wang, Xiangyao Sun, Tongtong Zhang, Xingyu Li.

**Formal analysis:** Wei Wang, Xiangyao Sun, Tongtong Zhang, Xingyu Li, Shibao Lu.

**Funding acquisition:** Shibao Lu.

**Investigation:** Wei Wang, Xiangyao Sun, Tongtong Zhang, Junzhe Ding, Shibao Lu.

**Methodology:** Wei Wang, Xiangyao Sun, Tongtong Zhang, Junzhe Ding.

**Project administration:** Wei Wang, Xiangyao Sun, Tongtong Zhang, Junzhe Ding.

**Resources:** Wei Wang, Xiangyao Sun, Siyuan Sun.

**Software:** Wei Wang, Xiangyao Sun, Siyuan Sun.

**Supervision:** Wei Wang, Xiangyao Sun, Siyuan Sun.

**Validation:** Wei Wang, Xiangyao Sun.

**Visualization:** Wei Wang, Xiangyao Sun, Chao Kong.

**Writing – original draft:** Wei Wang, Xiangyao Sun.

**Writing – review & editing:** Wei Wang, Xiangyao Sun.

Shibao Lu orcid: 0000-0002-2389-9683.

## Supplementary Material

Supplemental Digital Content

## Supplementary Material

Supplemental Digital Content

## Supplementary Material

Supplemental Digital Content

## Supplementary Material

Supplemental Digital Content

## Supplementary Material

Supplemental Digital Content

## Supplementary Material

Supplemental Digital Content

## Supplementary Material

Supplemental Digital Content

## Supplementary Material

Supplemental Digital Content

## Supplementary Material

Supplemental Digital Content

## Supplementary Material

Supplemental Digital Content
