# Supplementary figures and images for: Comparison between topping-off technology and posterior lumbar interbody fusion in the treatment of chronic low back pain: A meta-analysis
Source: Medicine (Baltimore). 2020 Jan 31;99(5):e18885. doi: 10.1097/MD.0000000000018885 (PMC7004705; doi:10.1097/MD.0000000000018885)

Supplementary File 1 Funnel plot of proximal RASP.


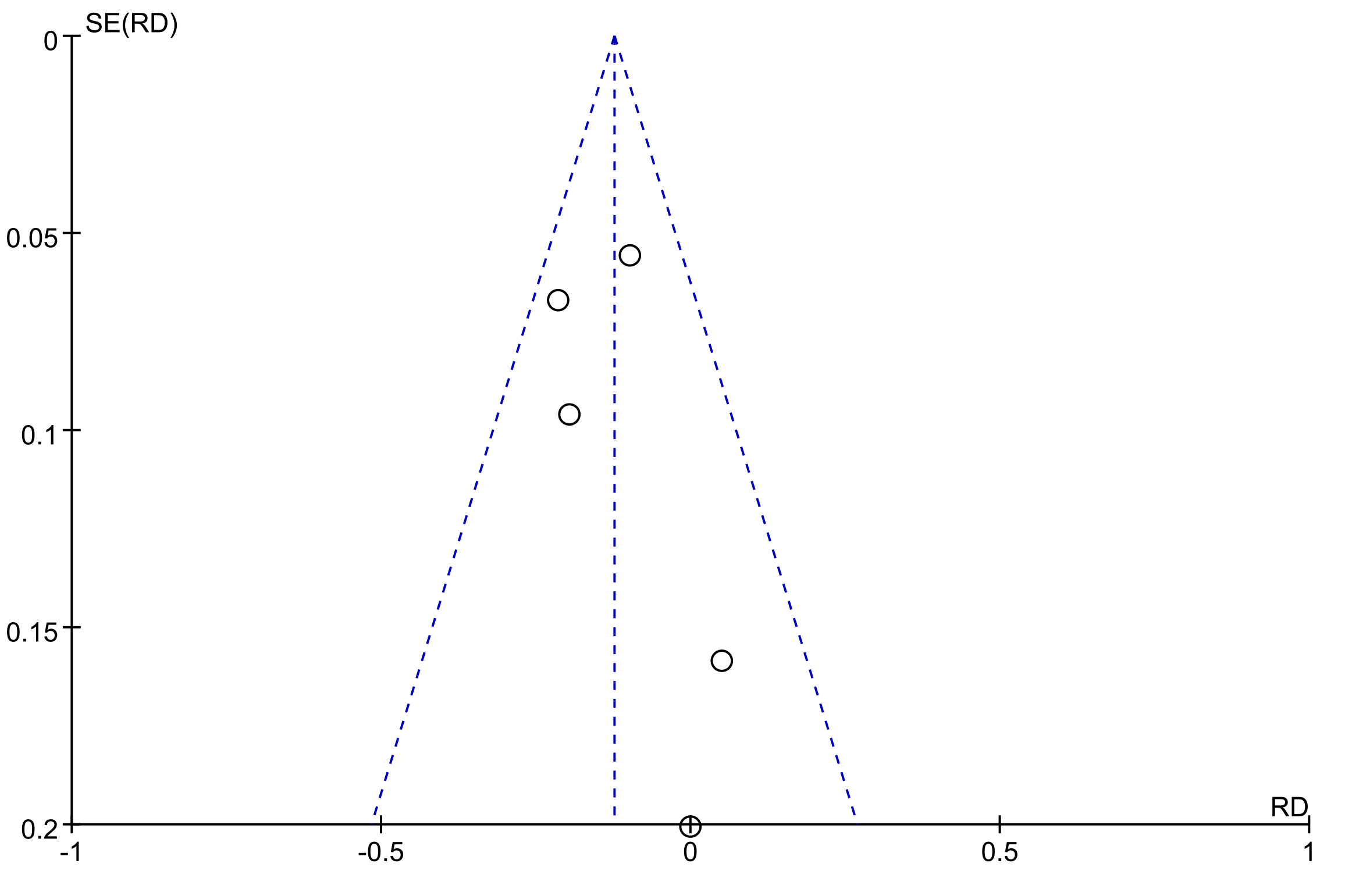

Supplement: Supplemental Digital Content [file medi-99-e18885-s001.doc]

Supplementary File 2 Funnel plot of CASP.


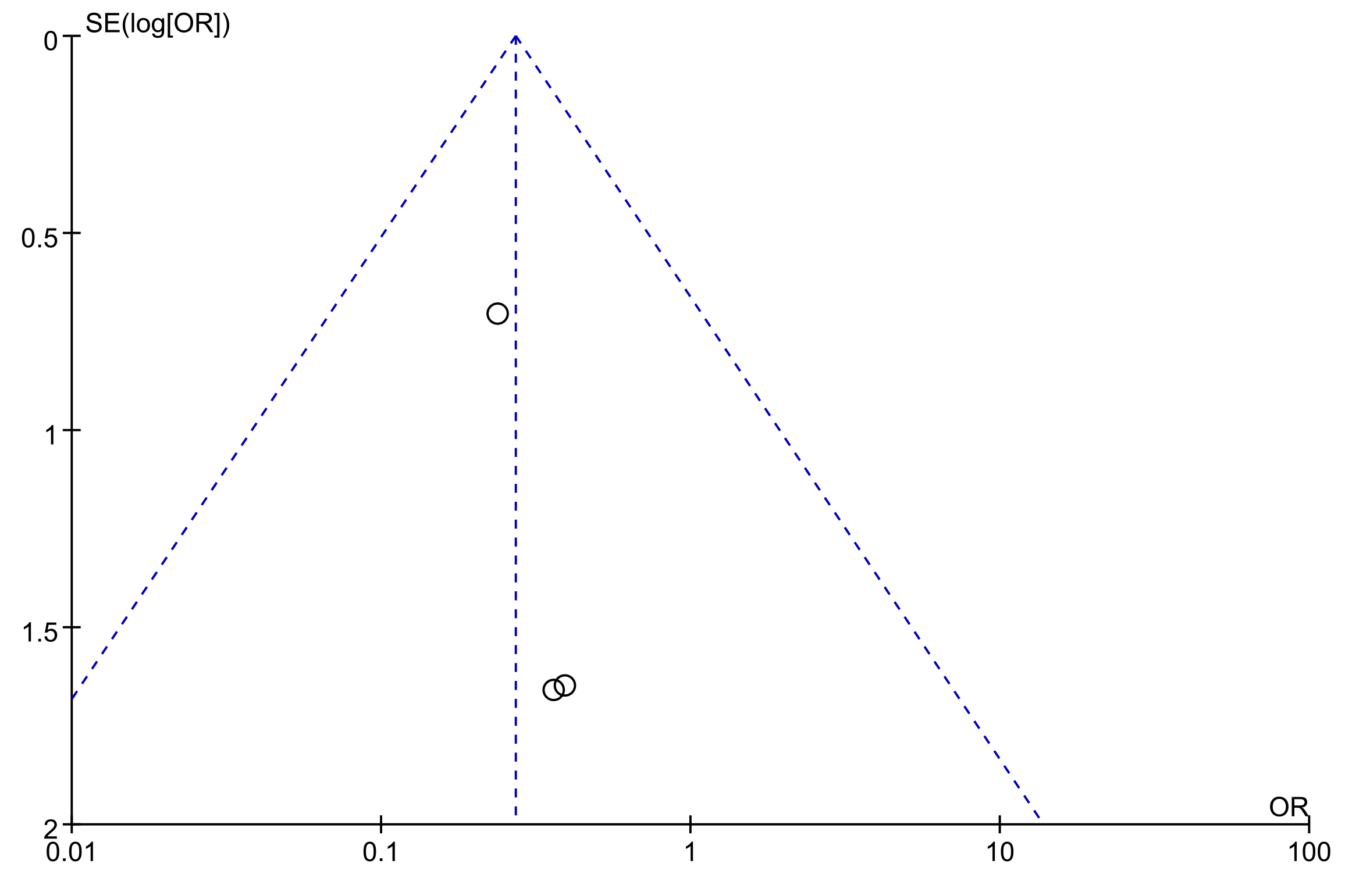

Supplement: Supplemental Digital Content [file medi-99-e18885-s002.doc]

Supplementary File 3 Funnel plot of postoperative GLL.


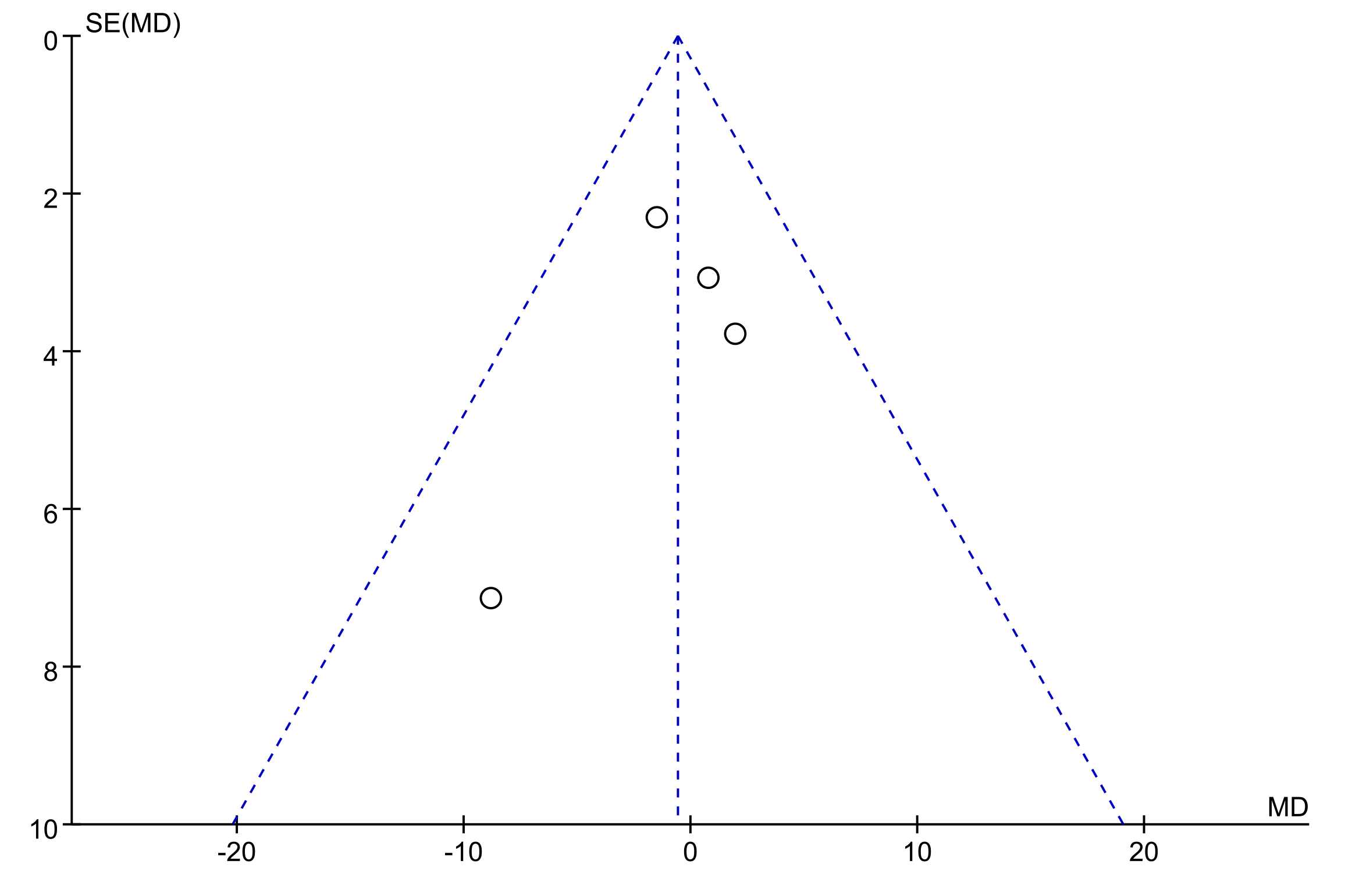

Supplement: Supplemental Digital Content [file medi-99-e18885-s003.doc]

Supplementary File 4 Funnel plot of postoperative VAS-B.


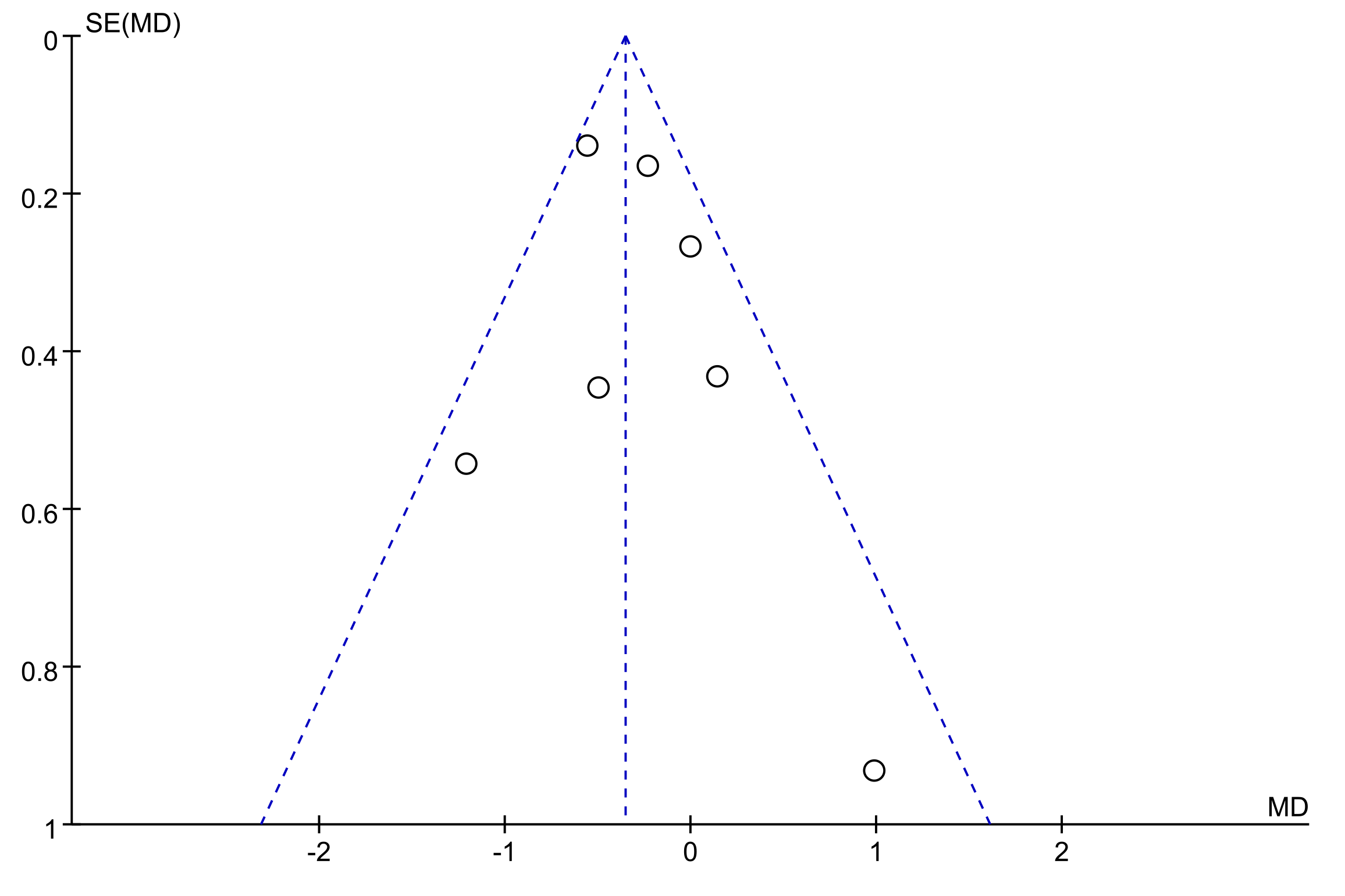

Supplement: Supplemental Digital Content [file medi-99-e18885-s004.doc]

Supplementary File 5 Funnel plot of postoperative VAS-L.


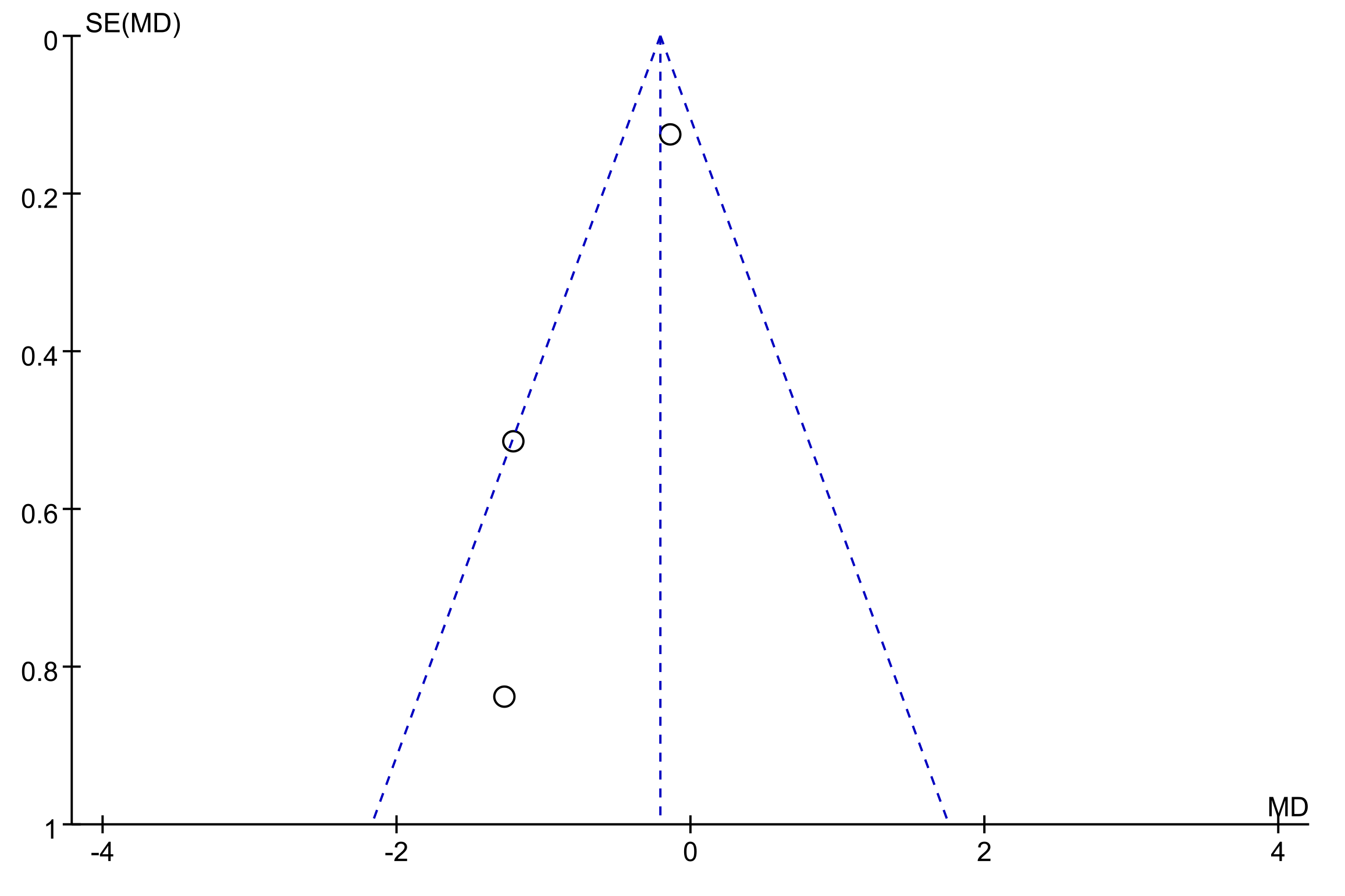

Supplement: Supplemental Digital Content [file medi-99-e18885-s005.doc]

Supplementary File 6 Funnel plot of postoperative ODI.


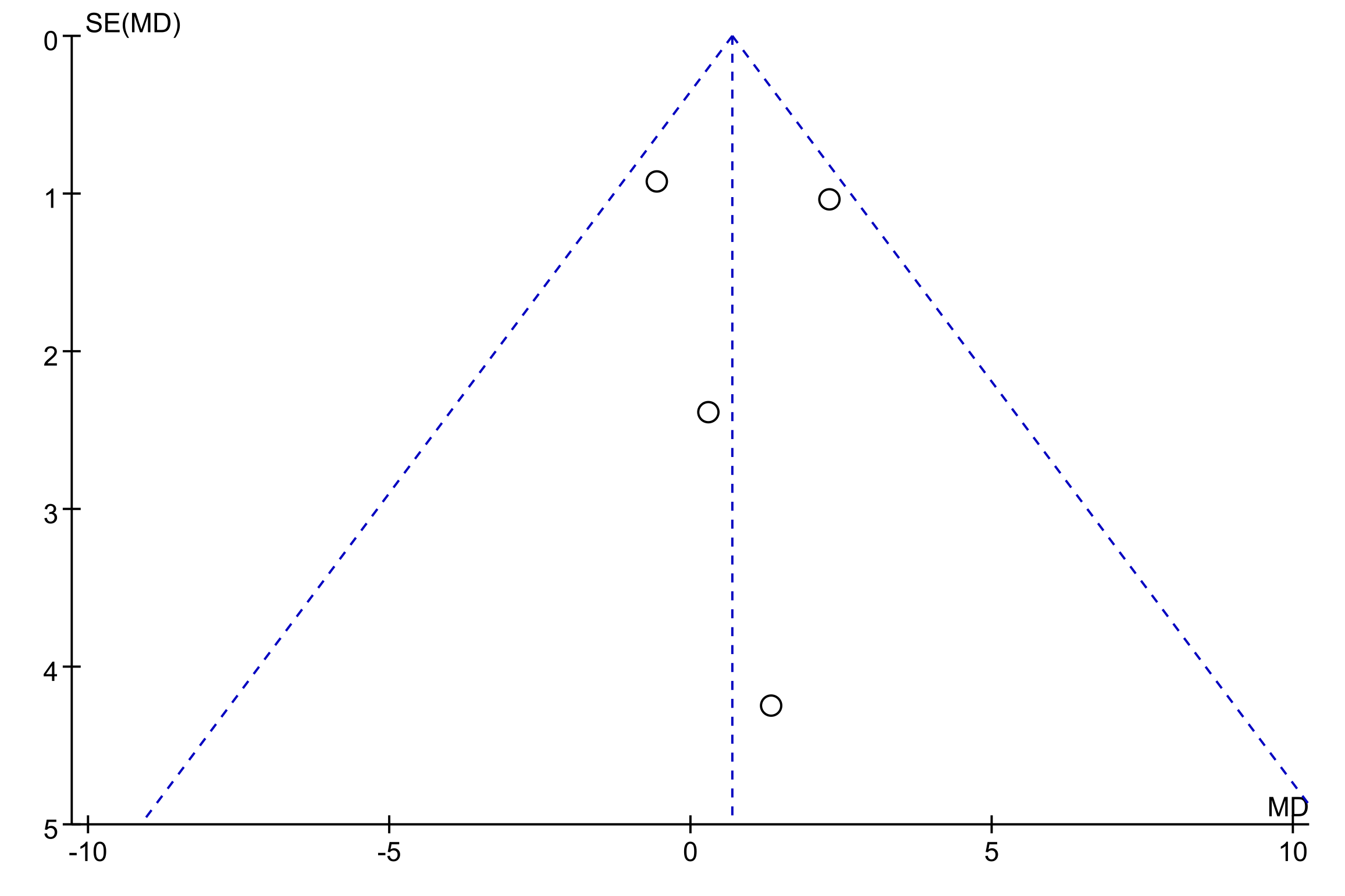

Supplement: Supplemental Digital Content [file medi-99-e18885-s006.doc]

Supplementary File 7 Funnel plot of complication rates.


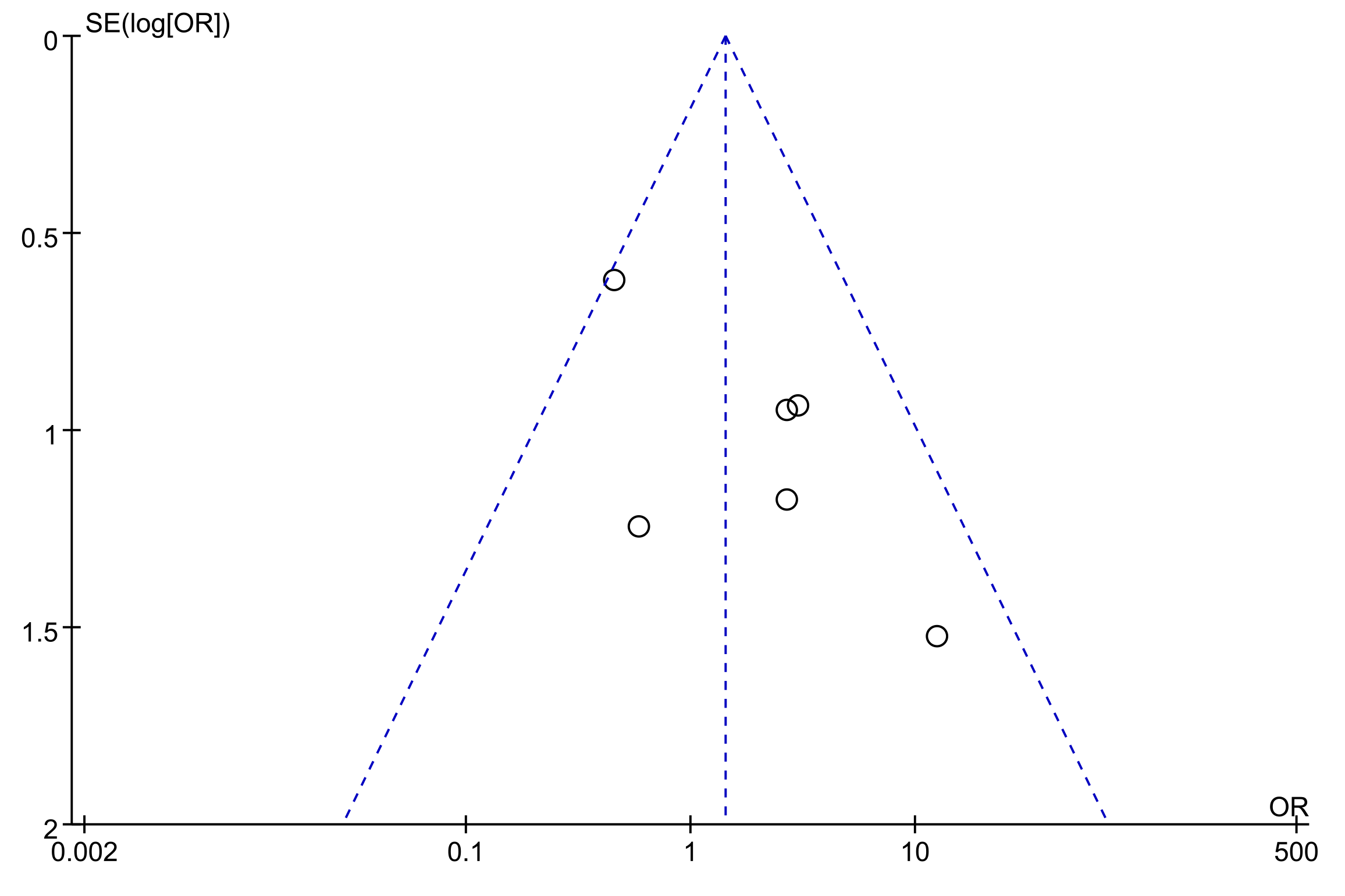

Supplement: Supplemental Digital Content [file medi-99-e18885-s007.doc]

Supplementary File 8 Funnel plot of infaction rates.


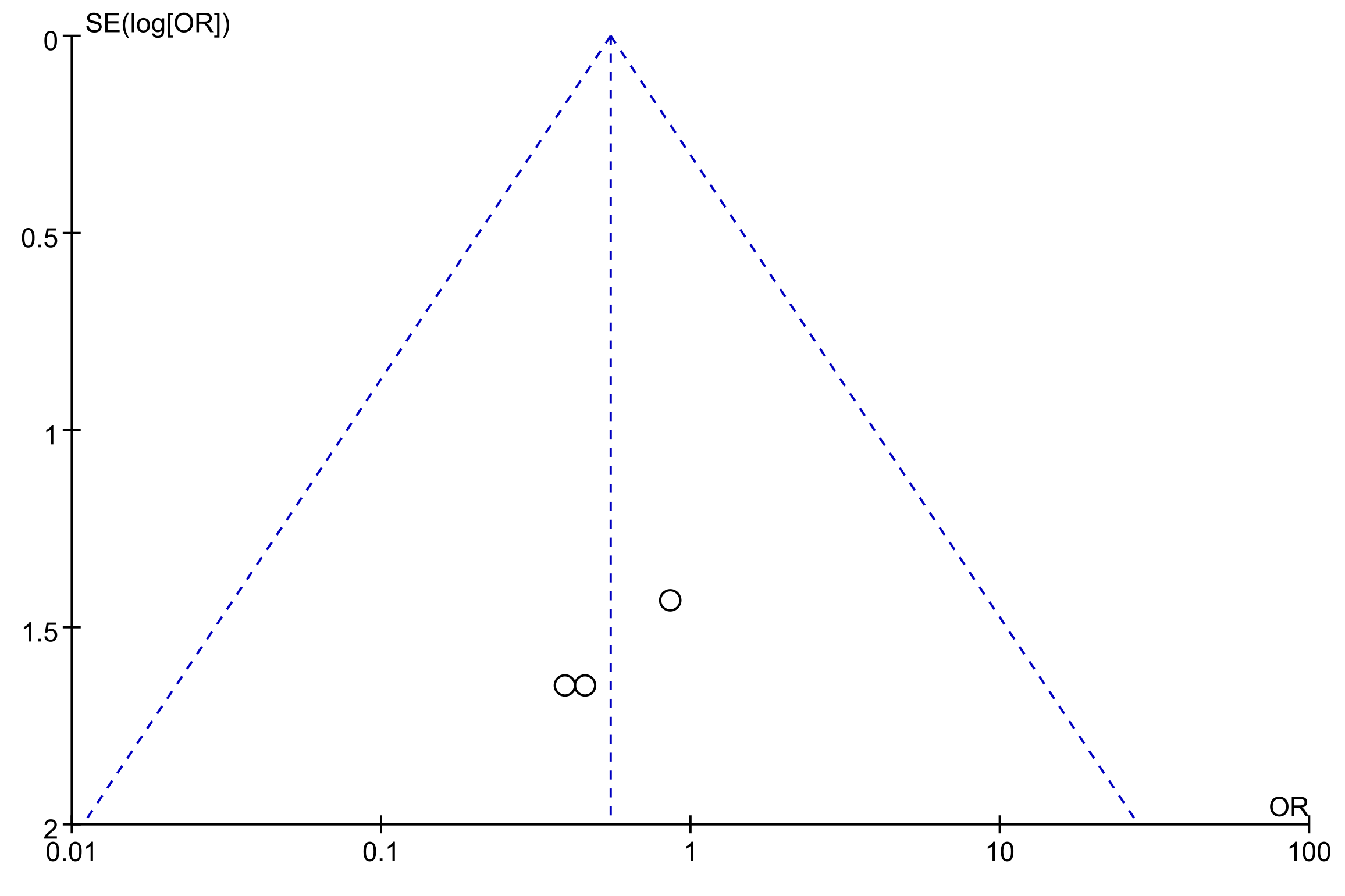

Supplement: Supplemental Digital Content [file medi-99-e18885-s008.doc]

Supplementary File 9 Funnel plot of reoperation rates.


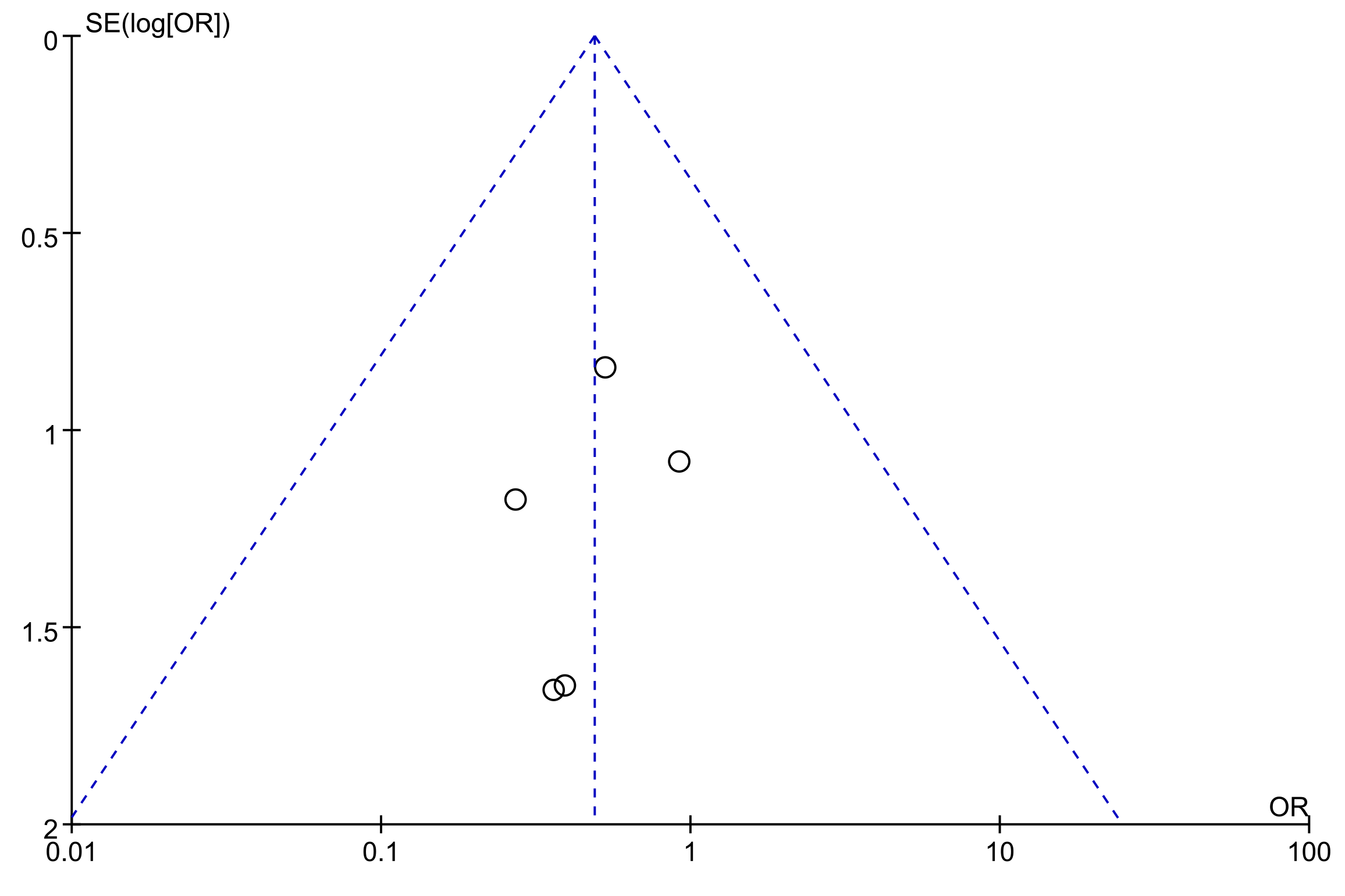

Supplement: Supplemental Digital Content [file medi-99-e18885-s009.doc]

Supplementary File 10 Funnel plot of gender.


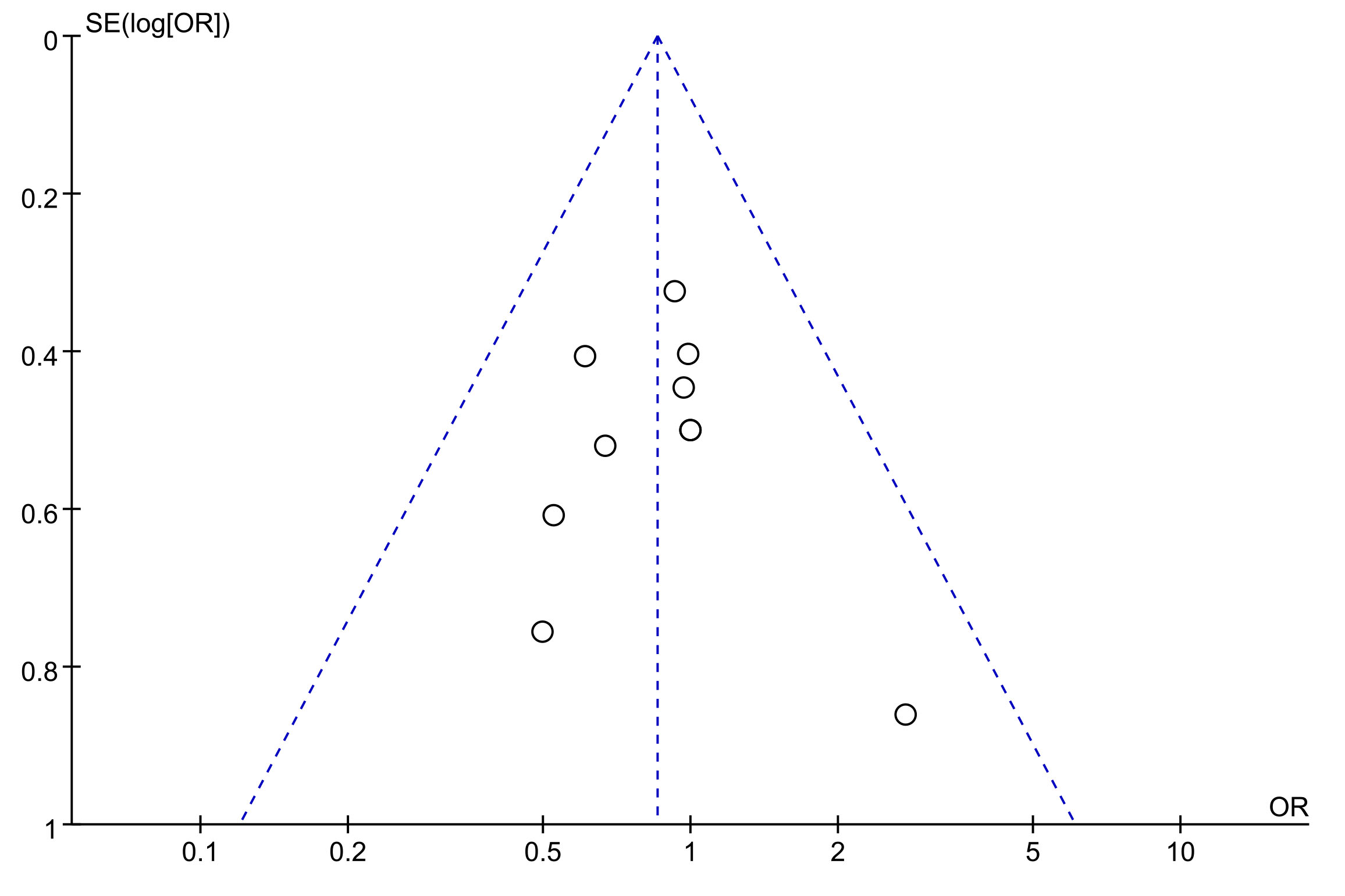

Supplement: Supplemental Digital Content [file medi-99-e18885-s010.doc]
